# Efficacy and safety of azithromycin and amoxicillin/clavulanate for otitis media in children: a systematic review and meta-analysis of randomized controlled trials

**DOI:** 10.1186/s12941-021-00434-x

**Published:** 2021-04-24

**Authors:** Gabriel Dawit, Solomon Mequanent, Eyasu Makonnen

**Affiliations:** 1 Center for Innovative Drug Development and Therapeutic Trials for Africa (CDT Africa), College of Health Sciences, Addis Ababa University, Addis Ababa, Ethiopia; 2 Department of Pharmacology and Clinical Pharmacy, College of Health Sciences, Addis Ababa University, Addis Ababa, Ethiopia

**Keywords:** Azithromycin, Amoxicillin/clavulanate, Otitis media, Meta-analysis, Systematic review

## Abstract

**Background:**

Resistance, prolonged therapy, and more adverse reactions made amoxicillin less preferred for treating otitis media. This study aimed to compare the efficacy and safety of azithromycin and amoxicillin/clavulanate for the treatment of otitis media in children.

**Methodology:**

This study was a systematic review and meta-analysis. PubMed, Cochrane library, and Google scholar databases were searched. Comparative randomized clinical trial studies between azithromycin and amoxicillin/clavulanate to treat otitis media in children published up to 30 September 2019 were included. The risk of bias was assessed and Data was extracted by the first author and checked by the second author. Meta-analysis was performed by STATA software version 16, and Mantel–Haenszel statistical method with effect measure odds ratio was employed for analysis.

**Result:**

751 records were identified and 14 studies were eligible for analysis. In 12 studies azithromycin had equivalent clinical efficacy and 2 had less to amoxicillin/clavulanate. Meta-analysis results showed no statistically significant difference in efficacy in favor of amoxicillin/clavulanate after completion of treatment OR 0.75, 95% CI (0.62–0.91). On subgroup analysis for children less than 2 years (OR 0.96 95% CI (0.49–2.29), and greater than 2 years (OR 1.40 95% CI (0.93–2.11) and also efficacy on follow up (OR 0.97 95% CI (0.83–1.15) there is no statistically significant difference. The clinical adverse events are more in the amoxicillin/clavulanate group than in the azithromycin with a statistical significant difference OR 0.46 95% CI (0.43–0.56).

**Conclusion:**

Azithromycin is comparable to amoxicillin/clavulanate to treat otitis media in children, and it is safer and more tolerable.

## Introduction

Otitis media is a middle ear infection, which exists in acute or chronic state and occur with or without symptoms caused by bacteria or virus [1]. About 80% of children have acute otitis media (AOM) once before the age of 3 years, and about 40% have six or more recurrences by the age of 3 years [2, 3]. Bacteria isolates 50% to 90% from middle ear fluid culture with cases of acute otitis media and otitis media with effusion [4]. The three most common bacteria which cause otitis media are *Streptococcus pneumoniae, Haemophilus influenzae*, *Moraxella catarrhalis* [5–7]. A systematic review done by DeAntonio et al. reported the incidence of otitis media under 6 years old from five countries; 9.2% in Nigeria, 10% in Egypt, 6.7% in China, 9.2% in India, 9.1% in Iran, and 7.8% in Russia [8]. Severe otitis media can cause medical complications, like mastoiditis, subperiosteal abscess, facial nerve palsy brain abscesses, meningitis, and chronic sinus infection [9, 10]. Otitis media have also an impact on childhood development: frequent occurrence of conductive hearing loss in less than 2 years of life has a negative effect on the development of the central auditory nerve pathways [11]. This effect results in deficits in auditory skills like attention, sound discrimination, the ability to listen for competing noise, and auditory memory [11]. These problems result delays in verbal language, intellectual development, and social skills.

Amoxicillin and other beta-lactam antibiotics considered standard treatments for otitis media are becoming less effective due to resistance emergence. Bacteria and viruses are responsible for otitis media and beta-lactams are active against bacteria only, but Azithromycin has antibacterial, antiviral, and anti-inflammatory activity. This study aimed to compare the efficacy and safety of azithromycin and amoxicillin/clavulanate for the treatment of otitis media in children from comparative randomized clinical trials through systematic review and meta-analysis.

## Methodology

### Study design and eligibility criteria

This study design is a systematic review and meta-analysis. The studies included are only randomized clinical trials conducted in children from 6 months to 15 years old with otitis media. Identified studies are included in the review if they met the following inclusion criteria:

1. A randomized clinical trial study for comparison of azithromycin and amoxicillin/clavulanate for treating any type of otitis media.
2. A randomized clinical trial study with clinical outcome cure and improve or failure from otitis media.
3. Participants of the study are children age ranged from 6 months up to 15 years.
4. Children with clinical evidence of bilateral or unilateral infection of the middle ear (OM).
5. Published article up to 30–09–2019.
6. A full-text article for review.
7. Articles written in English.

### Search strategy and data extraction

This review was designed according to the preferred reporting items for systematic reviews and meta-analysis protocols (PRISMA) guidelines [12]. Two databases (PubMed and Cochrane library) and manual search from Google Scholar were used to search the studies. Published research papers were systematically and comprehensively searched. All the search results from PubMed, Cochrane library and Google scholar were stored in Mendeley reference management software, and the duplicates were removed by the software. Both reviewers screened the title and abstract of each article depending on the PICOs criteria independently then checked for the eligibility criteria.

The data extraction form was developed with the help of the Cochrane collaboration data extraction form for interventional review for RCTs and Non-RCTs. All the available data were extracted with the extraction form. One author extracted the data from the included studies and the second author checked the extracted data. All disagreements were resolved by discussion between the two authors. The extracted data were stored in a Microsoft Excel spreadsheet.

### Data quality assessment

For each article, the following were critically appraised independently by two reviewers.

- Whether the study design or approach was appropriate to the research question.
- Whether outcome measure was valid and appropriate to the research question.
- The risk of bias in the study design and results were assessed by the Cochrane risk of bias tool-2 (ROB-2).

### Data analysis and synthesis

The findings of individual eligible and quality-assured studies were collated and summarized. Meta-analysis was performed using STATA software version 16 and Mantel–Haenszel statistical method and effect measure odds ratio was employed for data analysis, data synthesis, and creating tables (forest plot). Meta-analysis was performed for studies with a similar design on the same intervention and assessing same outcome and where sufficient data were available. The results were reported in an odds ratio with 95% CI in studies. Heterogeneity I^2^ was analyzed from the forest plot result and publication bias was also assessed by creating a funnel plot. We used a random and fixed effects model depending on the degree of heterogeneity between studies. Results, together with the associated interpretations and conclusions were generated from narrative and quantitative synthesis, and the review was presented on a table.

## Results

**Fig. 1:**
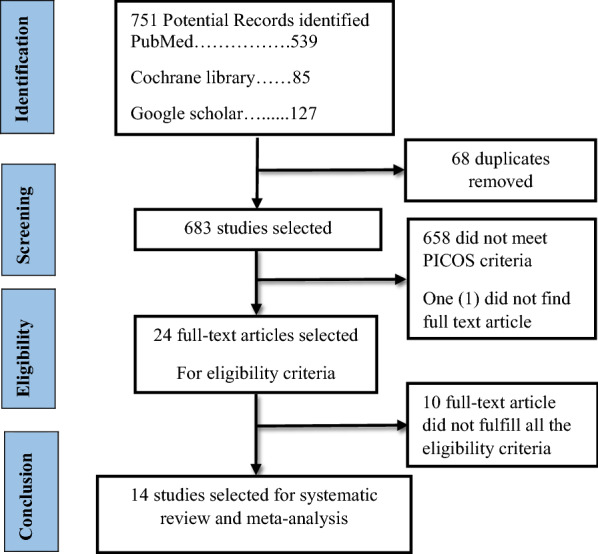
Study selection flow chart

From the three databases, 751 records, PubMed 539, Cochrane library 85, Google Scholar 127 (manual search) were identified. All search results were stored in Mendeley reference manager software. 67 duplicates were removed by the reference software Mendeley. In the first phase of title and abstract screening, 658 studies were excluded for not meeting PICOs criteria. Twenty four full-text articles full-filled the PICOs criteria out of which only 14 studies full filled the eligibility criteria to be included in the present systematic review and Meta-analysis. All the included studies were comparative randomized studies on azithromycin and amoxicillin/clavulanate of age range from 6 months to 15 years children. These 14 comparative RCTs were conducted in four continents and more than 22 countries with 226 centers of a total sample size of 5600 children. None of these studies was conducted in Africa and Australia and most were conducted in the US (Fig. 1).

### Data quality assessment

All the 14 studies were critically appraised independently by the two reviewers. In all 14 eligible RCTs, the study design and outcome measure was valid and appropriate to the research questions. The risk of bias in the study design and results were assessed by the revised tool Cochrane risk of bias in randomized trials (RoB 2) latest version 22 August 2019. The overall risk of bias of individual study is shown in Table 1. 8 studies were found to be low risk of bias and 6 were with some concern of bias but none of the studies was with a high risk of bias.

**Table 1:** Summary risk of bias of studies (ROBs-2)

S no.		Author (year)		Bias due to deviations from intended interventions	Bias from missing outcome data	Bias in the measurement of the outcome	Bias in selection of the reported results	The overall risk of bias
01		Daniel (1993)		Some concern	Lower risk	Lower risk	Lower risk	Some concerns
02		Schaad (1993)		Some concern	Lower risk	Lower risk	Lower risk	Some concerns
03		Principi (1995)		Some concern	Lower risk	Lower risk	Lower risk	Some concerns
04		Arguedasa et al (1996)		Lower risk	Lower risk	Lower risk	Lower risk	Low risk
05		Gerson (1996)		Some concern	Lower risk	Lower risk	Lower risk	Some concerns
06		Mohini (1996)		Some concern	Lower risk	Lower risk	Lower risk	Some concerns
07		Samuel (1996)		Lower risk	Lower risk	Lower risk	Lower risk	Low risk
08		Dagan (2000)		Lower risk	Lower risk	Lower risk	Lower risk	Low risk
09		Arrieta et al*.* (2003)		Lower risk	Lower risk	Lower risk	Lower risk	Low risk
10		Block et al. (2003)		Lower risk	Lower risk	Lower risk	Lower risk	Low risk
11		Dune et al. *(*2003)		Lower risk	Lower risk	Lower risk	Lower risk	Low risk
12		Hoberman et al. (2005)		Lower risk	Lower risk	Lower risk	Lower risk	Low risk
13		Guven et al. (2006)		Some concern	Lower risk	Lower risk	Lower risk	Some concerns
14		Arguedas et al. (2011)		Lower risk	Lower risk	Lower risk	Lower risk	Low risk

### Efficacy evaluation

**Fig. 2:**
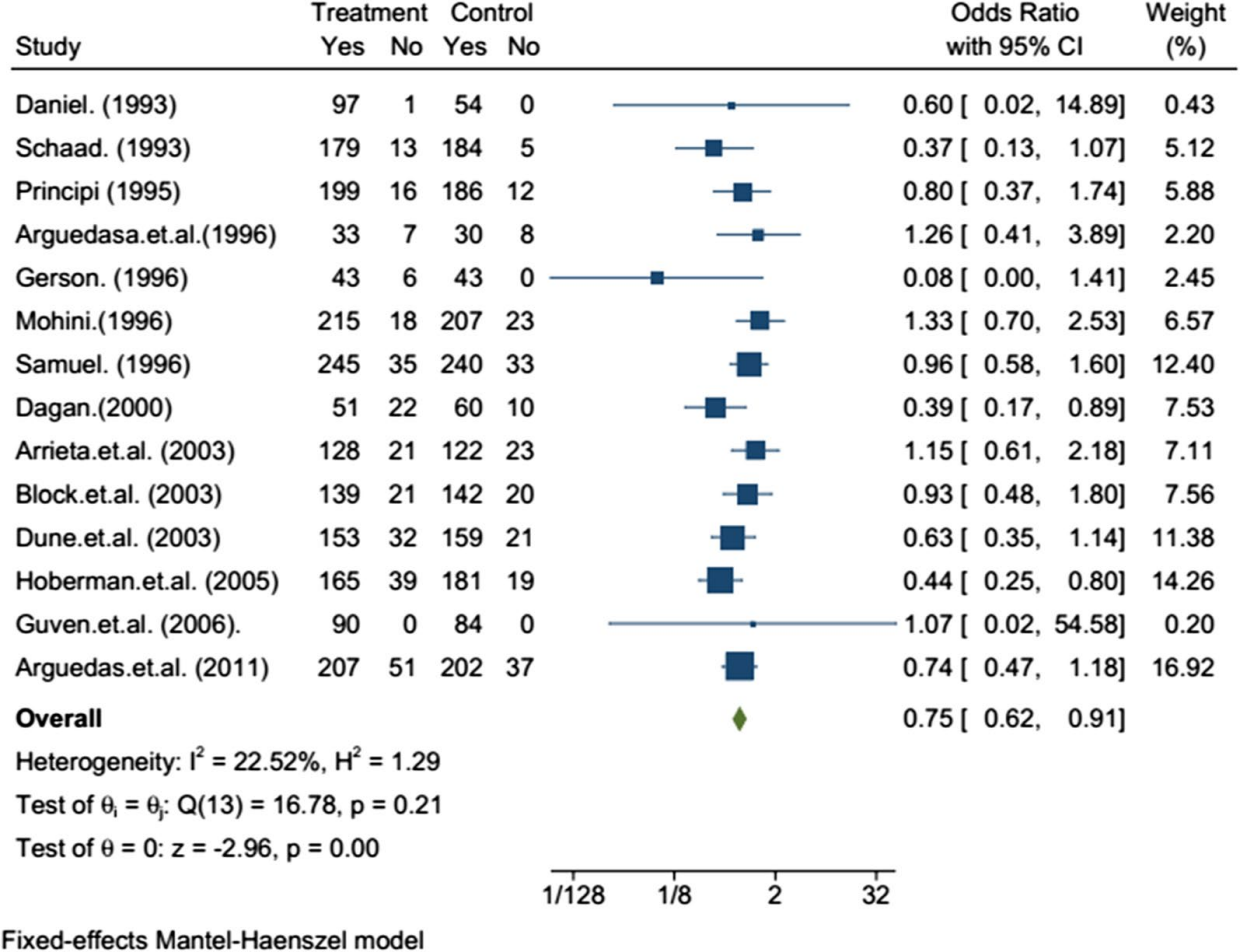
Forest plot: First efficacy assessment of study drugs with the estimates of OR and 95% CI

**Fig. 3:**
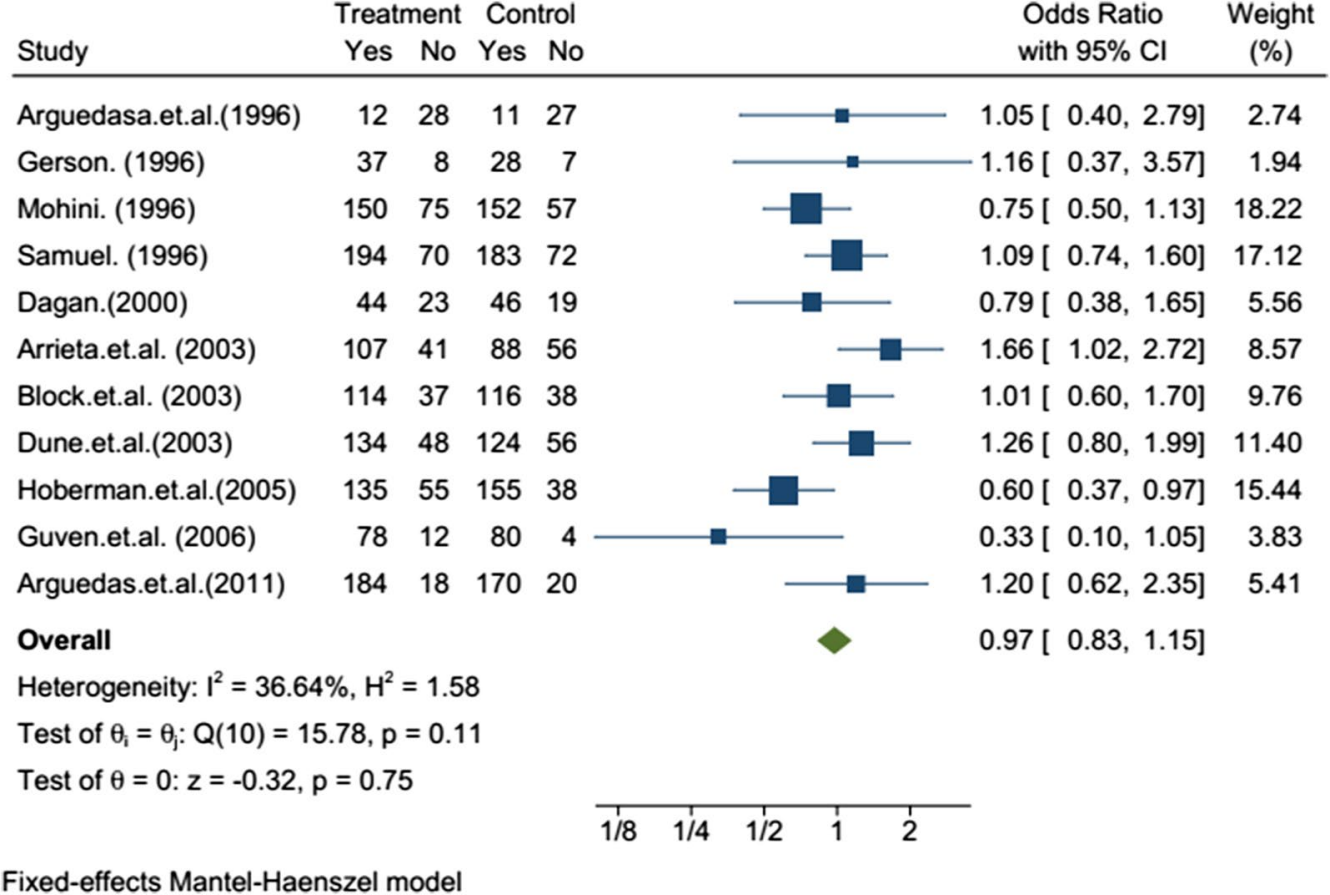
Forest plot: Second efficacy assessment of study drugs with the estimates of OR and 95% CI

**Fig. 4:**
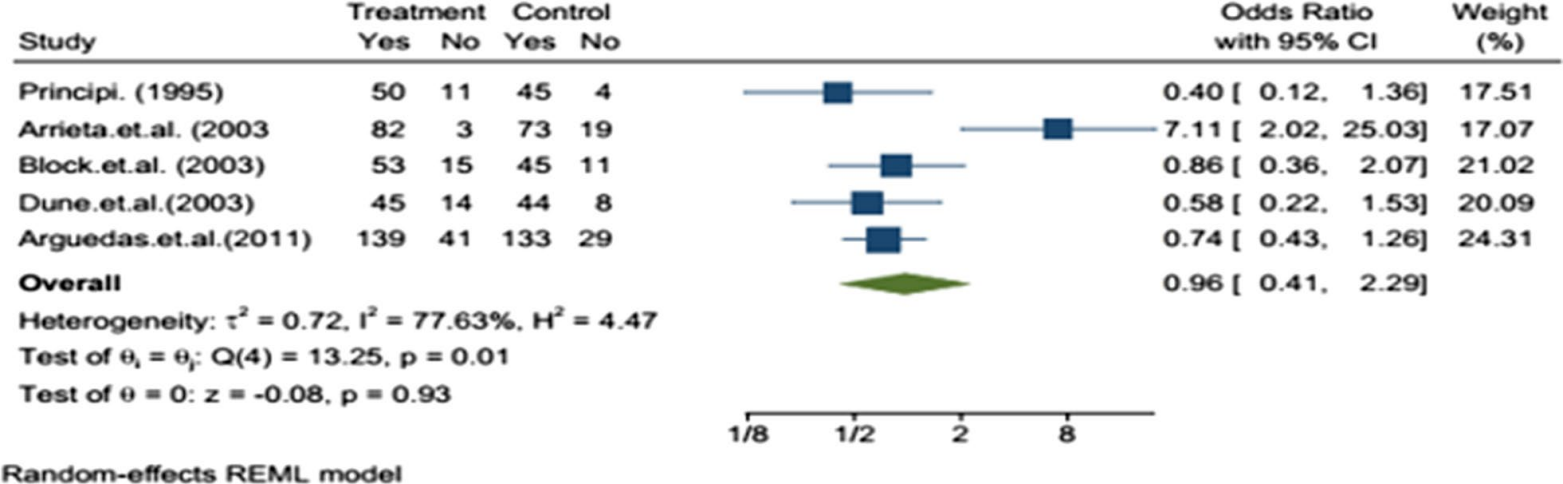
Forest plot: Efficacy assessment of study drugs with the estimates of OR and 95% CI in children less than 2 years

**Fig. 5:**
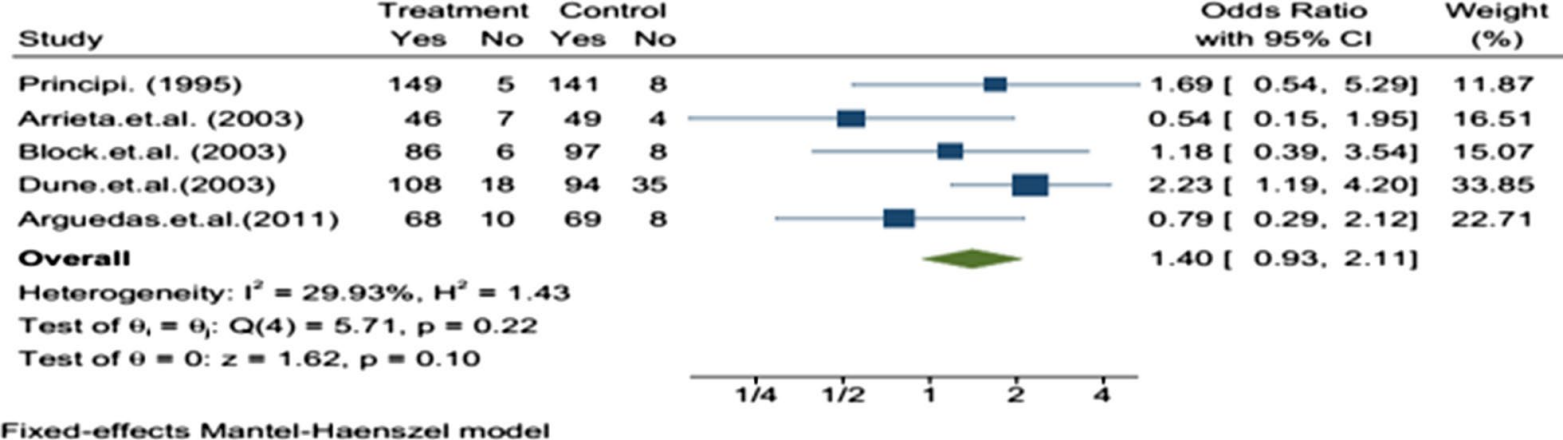
Forest plot: Efficacy assessment of study drugs with the estimates of OR and 95% in children greater than 2 years

The efficacy evaluation for fourteen studies after completion of treatment between the tenth to sixteenth days following initiation of treatment in the twelve studies, there was no statistically significant difference in the efficacy of azithromycin and amoxicillin/clavuluanate (Table 2). The efficacy assessment on long term follow-up between 3 and 5 weeks in eleven studies, the clinical [13–19] and bacteriological [20–22] efficacies were comparable with no statistically significant difference in ten of the studies, but the bacteriological efficacy of azithromycin was inferior to that of amoxicillin/clavulanate in one study [23]. The sub-group analysis was reported in five studies. In children less than or equal to two and greater than 2 years old, there was no statistically significant difference between azithromycin and amoxicillin/clavulanate groups in clinical [15, 18, 19, 24] and bacteriologic [22] efficacy. From the meta-analysis, the overall efficacy, i.e., both clinical and bacteriological efficacies, of the two treatments after completing treatment in the first assessment had little significant difference with OR = 0.75 95% CI (0.62–0.91) (Fig. 2) and on second assessment no significant difference with OR = 0.97; 95% CI (0.83–1.15) (Fig. 3). In subgroup meta-analysis, the overall effect between the groups in less than two and greater than 2 years children had no statistically significant difference with OR = 0.96; 95% CI (0.41–2.29) (Fig. 4) and OR = 1.40 95% CI [0.93–2.11] (Fig. 5) respectively.

**Table 2:** Included studies with their major findings

Author (year)	Target population age range (Children)	Study place	Sample size	Clinical success %Azithromycin/amoxicillin-clavulanate	P-value (95% CI)
Daniel (1993)	(2–8 year) with OM	Europe Multi center	159	99%/100%	NS (N/A)
Schaad (1993)	6 months to 12 years with AOM	Switzerland	389	93.2%/97.4%	NS (N/A)
Principi (1995)	(6-months to 12 years) with OM	Brazil, Chile, Germany, Italy, Korea, Spain, Turkey Venezuela	483	92.6%/93.9%)	NS (N/A)
Arguedasa et al*.* (1996)	6-months to 12 years with OM with effusion	San Jose Costa Rica	100	82.5%/78.9%)	NS (N/A)
Gerson (1996)	2 to 15 years with Acute otitis media	USA	169	87.7%/100%	0.102 (N/A)
Mohini (1996)	(6-months to 12 years) with Acute otitis media	USA	527	92.3%/90%	0.417 (N/A)
Samuel (1996)	1 to 15 years with AOM	USA	677	87.6%/87.9%	0.636 (N/A)
Dagan (2000)	6 months to 2 years with AOM	Israel USA and Dominican Republic	238	70% / 85.7%	0.023 (2,30)
Arrieta et al. (2003)	6-months to 6 years with recurrent or persistent AOM	USA, Latin American centers	304	85.9%/84.1%)	0.744 (− 6.4, 10)
Block et al. (2003)	(6-months to 12 years) with AOM	USA	350	86.9%/87.7%	NS (− 9.2, 6.5)
Dune et al*.* (2003)	(6-months to 12 years) with AOM	USA	373	82.7%/88.3%	0.186 (− 13, 3)
Hoberman et al. (2005)	6 month to 30 month with AOM	USA. Bulgaria, Chile, Domenica Republic, Guatemala, Israel, Peru, Romania Latvia Mexico,	731	80.9%/90.5%	< 0.01 (2.37,17)
Guven et al. (2006)	(6-months to 12 years) with AOM	Turkey	180	100%/100%	0.24 (N/A)
Arguedas et al (2011)	3 months–4 years with AOM	North America, Europe, and Latin America	923	80.2%/84.5%	0.24 (− 10.4, 2.6)

NS, non-significant; N/A, not-available; AOM, acute otitis media; OM, otitis media

### Clinical safety evaluation

**Fig. 6:**
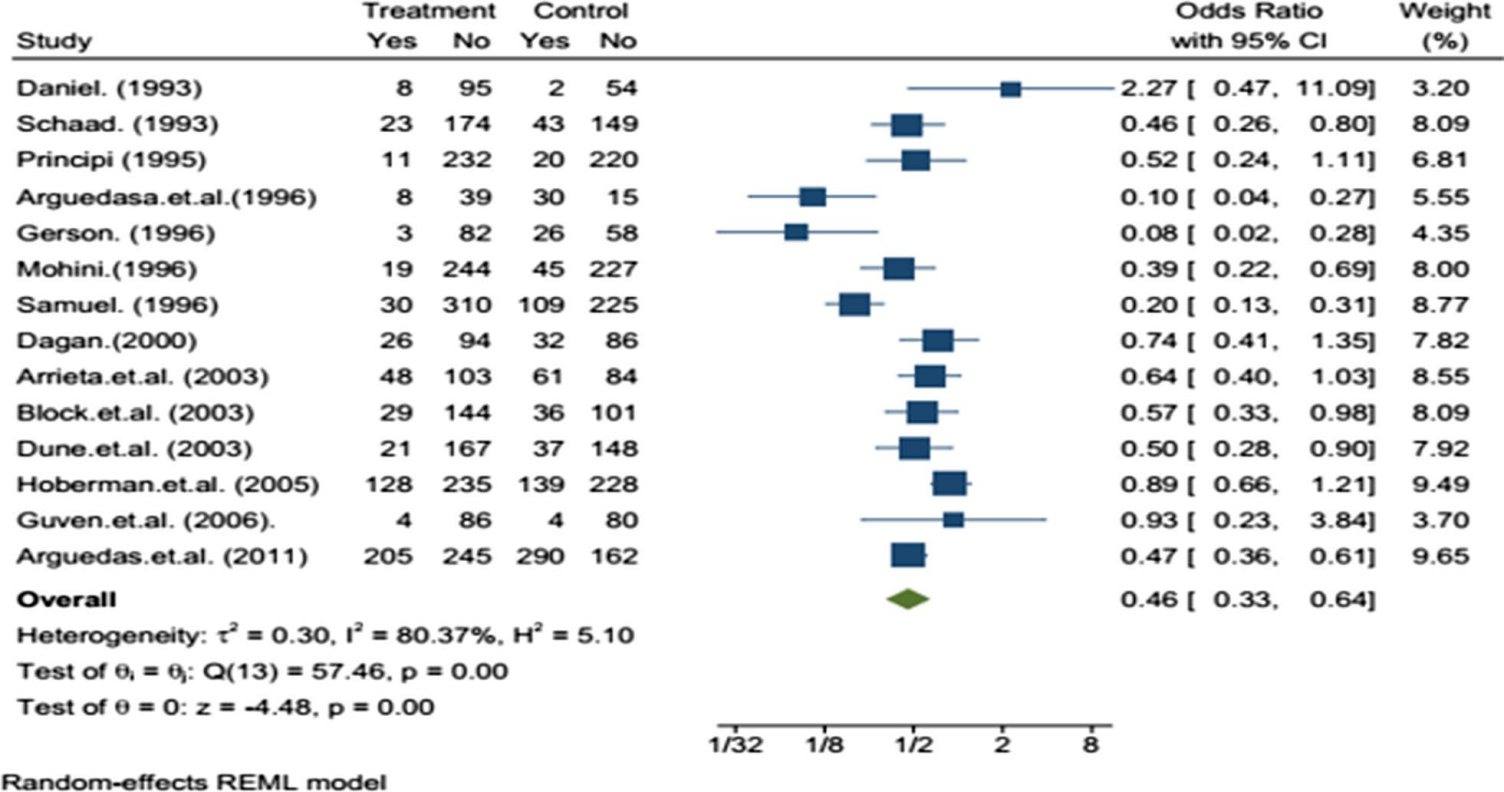
Forest plot: Clinical adverse events reported in the included studies with OR and 95% CI

Adverse effects of study drugs were reported as clinical adverse effects. Clinical adverse effects refer to the observable symptoms that occur after taking the medication. The incidence of clinical adverse effects of amoxicillin-clavulanate was significantly higher than that of azithromycin (Table 3). The most common clinical adverse effects observed with both drugs were gastrointestinal disorders (diarrhea, vomiting, nausea, abdominal pain, and loose stool), skin rash, and fever. The meta-analysis on clinical adverse effects showed a statistically significant difference with OR = 0.4695% CI (0.33–0.64) in favor of amoxicillin/clavuluanate (Fig. 6).

**Table 3:** Clinical adverse effects of study drugs in children

Author (year)	Azithromycin group	Amox-clav group	P-Value
Daniel (1993)	7.77%	3.7%	N/A
Schaad (1993)	11.7%	22.4%	< 0.002
Principi (1995)	4.5%	8.3%	0.0146
Arguedas et al. (1996)	17.%	66.7%	N/A
Gerson (1996)	3.5%	31%	< 0.001
Mohini (1996)	7.2%	17.11%	< 0.001
Samuel (1996)	8.8%	32.64%	< 0.0001
Dagan (2000)	21.7%	27.12%	0.327
Arrieta et al. (2003)	31.8%	42.07%	0.095
Block et al. (2003)	16.8%	22.54%	NS
Dune et al. (2003)	11.2%	20%	0.014
Hoberman et al. (2005)	35.3%	37.9%	NS
Guven et al. (2006)	4.4%	4.76%	NS
Arguedas et al. (2011)	45.6%	64.16%	N/A

### Publication bias

**Fig. 7:**
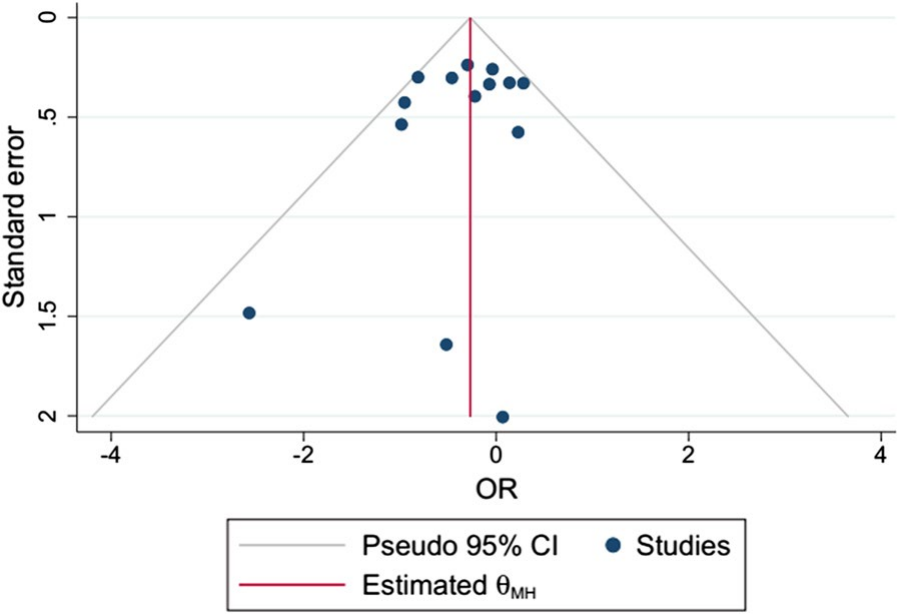
Funnel plot for publication bias assessment of included studies

No publication bias was reported as shown in the funnel plot from STATA.16 depicted in Fig. 7. The included studies are systematically distributed which shows an absence of bias. Three studies have a small size widely scattered at the bottom of the graph with less treatment effect estimation.

## Discussion

In this systematic review and meta-analysis, 14 studies were included. The efficacy evaluation was either clinical or bacteriological efficacy. The clinical efficacy of both study drugs was equivalent with no statistically significant difference in 12 studies. The bacteriological efficacy evaluation of amoxicillin/clavulanate was superior to azithromycin [16, 23], but in one of these studies, the dose of amoxicillin was doubled to 90 mg/kg while that of azithromycin not and in children with no otitis media pathogens there is no significant difference in the clinical efficacy of both drugs [23]. In two studies in all the patients with bacteriological culture-positive test, there is no difference in bacteriologic efficacy of both treatment groups [20, 23]. In a meta-analysis of a similar study, the bacteriologic efficacy of azithromycin was higher on the treatment of some bacterial respiratory infections on children (OR = 0.78, 95% CI (0.65–0.93), P = 0.007) than that of amoxicillin/ clavulanate [25]. In another similar study which compared 5 days of azithromycin dose, a single intramuscular dose of ceftriaxone and 10 days of amoxicillin/clavulanate dose for treatment of acute otitis media had shown all the equivalent efficacy with 87.1%, 85.3%, and 87.2% respectively [26]. The failure rates of amoxicillin alone (50 mg/kg twice a day for 7 days) and azithromycin (single dose 30 mg/kg) for treatment of acute otitis media were 54% (83/155) and 50% (82/165) respectively in another study [27]. In a meta-analysis of 20 RCTs assessed the clinical efficacy of azithromycin and amoxicillin/clavulanate for treatment of upper respiratory infection (acute otitis media and others) in 4980 children, the difference in efficacy was not significant with OR = 0.75 95% CI (0.62 0.91) P-value 0.003 though the trend was higher with azithromycin [25], while in our meta-analysis the efficacy of both study drugs was comparable with OR = 0.75 95% CI (0.62–0.91), but the trend was higher with amoxicillin/clavulanate.

The follow-up efficacy analysis on 21–35 days after treatment initiation was done in 11 studies. In 9 of the studies, the clinical efficacy was not significantly different in both treatment groups. The bacteriological efficacy of amoxicillin/clavuluanate in a single study showed better compared to azithromycin, while no significant difference was observed in the other 3 studies**.** The difference was smaller than on the first efficacy assessment. In both treatment groups, the efficacy was decreased in comparing with the first efficacy assessment. The efficacy of azithromycin was higher in 6 studies**,** lower in 4 studies, and equal in one study compared with amoxicillin/clavuluanate. The meta-analysis had shown the efficacy of azithromycin and amoxicillin/clavuluanate on day 21–35 is equivalent with OR = 0.97; 95% CI (0.83–1.15).

The subgroup analysis on the efficacy was also evaluated in less or equal to two and greater than 2 years old children. There was no statistically significant difference in all included 5 studies. The meta-analysis result in less or equal to two and greater than 2 years old children was OR = 0.96; 95% CI [0.41–2.29] and OR = 1.40 95% CI [0.93–2.11], respectively. The efficacy of both drugs was a bit higher in children greater than 2 years old children.

Azithromycin is safer and tolerable than amoxicillin/clavuluanate. The clinical adverse events found in both groups were similar. In our meta-analysis of 14 studies, clinical adverse events were higher with amoxicillin/clavuluanate with OR = 0.4695% CI (0.33–0.64). A similar meta-analysis done on 13 RCTs that assessed the safety of azithromycin and amoxicillin/clavuluanate in children with bacterial respiratory infections showed azithromycin was safer with statistically significant difference (OR = 0.49, 95% CI (0.40, 0.60), P < 0.000 01) [25] which was quite similar. In another study conducted in children of Australia and New Zealand the adverse effects observed with azithromycin was 17 (21%) of 82 while with amoxicillin–clavuluanate it was 23 (24%) of 97 (RR = 0·9, 95% CI 0·5 to 1·5) [28]. Also, a study by Ferwerda.*et.al* reported a higher incidence of adverse effects in amoxicillin/clavuluanate than azithromycin 43% versus 19%, respectively [29]. The most clinical findings observed in both treatment groups were gastrointestinal problems such as vomiting, diarrhea, nausea, and abdominal cramp.

## Conclusion

From the present systematic review and meta-analysis, it can be concluded that the efficacy of azithromycin is comparable to amoxicillin/clavulanate, and it is safer and more tolerable by children. Azithromycin can, also be considered a drug of choice in treatment of otitis media on children.

## Data Availability

The sources of data for this systematic review and meta-analysis research are from PubMed, Cochrane library, Google scholar. All the data are available in the following research articles: Arguedas et al. [22] Single-dose extended-release azithromycin versus a 10-day regimen of amoxicillin/clavuluanate for the treatment of children with acute otitis media International Journal of Infectious Diseases 15; e240–e248 https://doi.org/10.1016/j.ijid.2010.12.003. Arguedas et al. [13] Comparative trial of 3-day azithromycin versus 10-day amoxicillin/clavuluanate potassium in the treatment of children with otitis media with effusion. International Journal of Antimicrobial Agents 6; 233–238. Arrieta and Singh [6] Management of recurrent and persistent acute otitis media: new options with familiar antibiotics. Pediatric Infectious Disease Journal; 23 (2):115–124. Block et al. [19] Single-dose (30 mg/kg) azithromycin compared with 10-day amoxicillin/clavuluanate for the treatment of uncomplicated acute otitis media: a double-blind, placebo-controlled, randomized clinical trial. Current Therapeutic Research 6 4: A30-A40. https://doi.org/10.1016/j.curtheres.2003.09.006. Dagan et al. [21] Bacteriologic and clinical efficacy of amoxicillin/clavuluanate *vs.* azithromycin in acute otitis media. Pediatric Infectious Diseases Journal. 19 (2):95–104. Daniel et al. [30] Comparison of azithromycin and co-amoxiclav in the treatment of otitis media in children Journal of Antimicrobial Chemotherapy. 31, E, 65–71. Gerson [20]. A multicenter open label trial of azithromycin versus amoxicillin/clavulanate for the management of acute otitis media in children. Pediatric Infectious Diseases Journal 15 (6) 15–19. Guven et al. [16] Bacterial etiology of acute otitis media and clinical efficacy of amoxicillin—clavuluanate versus azithromycin. International Journal of Pediatric Otorhinolaryngology: 70: 915—923. Hoberman et al. [23]. Large dosage amoxicillin/clavuluanate, compared with azithromycin, for the treatment of bacterial acute otitis media in children. Pediatric Infectious Diseases Journal. 24 (6): 525–532. https://doi.org/10.1097/01.inf.0000164794.50281.1a. Mohini [17]. A multicenter randomized open-label comparison of azithromycin and amoxicillin/clavuluanate in acute otitis media among children attending daycare or school. Pediatric Infectious Diseases Journal. 15 (09): 24–29. Principi [24]. Multicenter comparative study of the efficacy and safety of azithromycin compared with amoxicillin/clavulanic acid in the treatment of pediatric Patients with Otitis Media. European Journal of Clinical Microbiology and Infectious Diseases. 14 (8):669–676. Samuel [14]. A multi-center double blind comparison of azithromycin and amoxicillin- clavuluanate for the treatment of acute otitis media in children. Pediatric Infectious Diseases Journal. 15(9):20–23. Schaad [31]. Multicenter evaluation of azithromycin in comparison with co-amoxiclav for the treatment of acute otitis media in children. Journal of Antimicrobial Chemotherapy: 31: 81–88.
